# The Properties of Lightweight Aggregates Pre-Coated with Cement Pastes and Their Suitability for Concrete

**DOI:** 10.3390/ma14216417

**Published:** 2021-10-26

**Authors:** Lucyna Domagała, Ewa Bryła

**Affiliations:** Faculty of Civil Engineering, Cracow University of Technology, 31-155 Cracow, Poland; ebryla@wp.pl

**Keywords:** lightweight aggregate, aggregate impregnation, sintered fly ash, expanded clay, lightweight concrete, oven dry density, water absorption, compressive strength

## Abstract

One of the biggest technological problems connected with the production of lightweight concretes made of porous aggregates is their much higher water absorption, which may cause on the one hand workability loss, and on the other hand excess water content in concrete. The aim of this research was to assess the effect of impregnation of lightweight aggregates (LWAs) with cement paste on their properties and to verify its effectiveness in concretes. Three types of lightweight aggregates differing in porosity and pore structure (sintered fly ash Lytag, expanded clay: Leca and Liapor) were selected for the tests. The following parameters were taken into consideration in the research program: LWA type and size, LWA initial moisture content, strength, and rheology of cement pastes. The tests of 22 different aggregates, plain and coated with cement paste, included density, crushing strength, and development of water absorption in time. The research program proved that porous aggregates, due to their impregnation with cement pastes, may be effectively sealed and strengthened. All tested LWAs showed a considerable decrease in water absorption by up to 71%. However, only Lytag aggregate showed a visibly enhanced crushing strength. Verification of effectiveness of aggregate sealing on the enhancement of concrete properties showed both a considerable reduction in water absorption of composites (by up to 52%) and a very high increase in their strength (by up to 107%).

## 1. Introduction

Lightweight aggregate concrete (LWAC) is deemed as one of the most comprehensive building materials, used for both structural and insulating purposes, and made for both monolithic and precast applications. Due to its lower density, it is possible to construct higher buildings, structures, and elements of longer spans and/or smaller cross-sections to build constructions on poorer grounds [1,2,3,4]. On the other hand, owing to the application of by-products for aggregate manufacturing in better thermal insulation, possible better durability [5,6,7,8,9], and possibility of recycling, LWAC is regarded as one of the most sustainable building materials. Despite all above advantages, lightweight aggregate concrete is still less popular than normal-weight concrete (NWAC). Besides the typically higher cost of LWAC volume unit, the second reason of this situation is the more complicated technology involved in its production.

Application of lightweight aggregate (LWA) may increase the risk of fresh concrete segregation and/or workability loss, resulting from much lower LWA particle density (typically from 400 up to 2000 kg/m^3^) and much higher LWA water absorption (typically from 5 up to 45%) in relation to normal-weight aggregate (NWA) [4]. It should be clearly stated that the absorption of water by lightweight aggregate immersed in water and in fresh concrete differs. It was estimated that LWA in concrete mixture may absorb about 60 up to 100% of water, which would be able to be absorbed by the aggregate submerged in pure water [3,10,11]. However, in the case of very low water-binder ratio (0.30), this share may be assessed as low as 20–50% [12]. Water absorbed by the lightweight aggregate from 1 m^3^ of fresh concrete is estimated at 30–90 kg [10] or even 70–100 kg [3,4]. As it was proved in [13,14], the extent of the water-cement ratio (w/c) reduction resulting from lightweight aggregate water absorption in concrete depends on LWA initial moisture content, LWA content in concrete, and the rheological characteristics of the cement matrix. When initially dry sintered fly ash aggregate was used, the water-cement ratio could be reduced even by up to 35% (by up to 0.20). While the same aggregate initially wetted with a moisture content of 17% was able to reduce w/c of fresh concrete at most by up to 14% (by up to 0.05) [13].

To protect LWAC mixture against its workability loss and segregation, lightweight aggregate is usually pretreated prior to use. The most popular treatment is pre-wetting LWA particles. Alternatively, additional water content determined by LWA water absorption is included in the concrete composition. The water content extra dosed for concrete mix or used for aggregate pre-wetting is established at different levels, corresponding to LWA water absorption after 1–2 h (e.g., [9,15]), 24–48 h (e.g., [16,17]), or even more (e.g., [5,7]). As a result, in many cases the aggregate is used for concrete fully saturated with water. Such treatments are certainly the most effective for the elimination of segregation and workability loss risks. Since these procedures are very effective as well as cheaper and simpler than the others, pre-wetting is nowadays commonly used in practice.

Nevertheless, there are many research reports showing some negative aspects of aggregate pre-saturation. Concretes containing LWA of higher water absorption (WA_24h_ ≥ 10–15%), which were initially pre-saturated or pre-wetted to a high moisture content may reveal: lower strength [9,18,19], higher water permeability [9,19], lower freeze-thaw resistance [5,7,9], deeper carbonation [8,16], and deeper penetration of chloride ions [8,16]. Moreover, research focused on LWAC microstructure [19,20] showed that the interfacial transition zone (ITZ) of LWAC prepared with pre-wetted or pre-saturated aggregates revealed a poorer bond with a bigger number of portlandite crystals, a higher content of ettringite, and assisting microcracks.

The alternative method to eliminate workability loss and segregation risk is to impregnate LWA. There are many technologies used for aggregate impregnations. In order to increase the aggregate tightness, depending on its type, it may be coated with e.g.,: cement paste [21,22,23,24,25,26], natural and synthetic polymers [21,24,27,28,29,30,31,32], calcium metasilicate [33], sodium silicate [22,24,30] or sodium carbonate solutions [34,35], kerosene, different oils [24], paraffin [18,31,36,37,38,39]. It should be stated that in some research, the priority goal of LWA immersion in paraffin or polyethylene glycol is not so much to make concrete tighter but to incorporate the phase change material into the composite in order to improve its thermal properties [31,36,37] or freeze-thaw resistance [39]. Similarly, in the case of pre-coating lightweight aggregates with silicates or carbonates solutions, apart from the immediate sealing effect, the self-healing mechanism is also important [30,34,35]. Generally, the process of pre-coating for natural or manufactured LWA of mineral origin is much simpler in relation to procedures applied for recycled aggregates or natural organic ones. In this last case before impregnation, sometimes it is recommended to enhance weak surface of aggregate particles by additional treatments e.g., soaking in acid, in silica fume or nanosilica solution, calcic lime solution, or cement paste [18,21,33,40]. In the case of typical LWA for structural concretes, there is no need to improve the aggregate bond.

Generally, the impregnation process of a lightweight aggregate may lead to a lower cement content, stabilization of concrete workability, decrease in concrete water absorption, shorter time of element/structure drying out, the increase in concrete durability, strength, thermal, and acoustic insulation [24,25,28,29,30,31,32,33,36]. On the other hand, LWA pre-coating may affect the higher concrete density, lack of internal curing with water accommodated in aggregate, higher autogenous shrinkage, and higher rate of drying shrinkage, leading to a worse bond between aggregate and cement paste [24,31,33]. It should be stated that qualitative and quantitative effectiveness of LWA impregnation is strongly determined by the type of aggregate as well as the type of impregnant and procedure of pre-coating, since these parameters dictate the mechanism of cement paste absorption. Research [41,42] has shown that some expanded clay aggregates with strongly sintered external shells do not reveal the ability to absorb cement paste from fresh concrete at all. Only the surface pores of these aggregates were tightened. The amount of cement paste absorbed by such LWA types was estimated in [43] for 1.0–2.5% of aggregate mass in an oven dry condition. The aggregates of less sintered shells with open pores are more prone to the ingress of cement paste. The depth of cement paste migration into the structure of porous aggregate is regarded by some researchers [20] as dependent on LWA initial moisture content, while others [19,41] indicated that it is independent. It should be stated that the cement paste migration into the aggregate consists not only of direct cement paste absorption, but mainly of the migration of dissolved cement particles into the aggregate pores filled with pre-absorbed water. That is why in the research reported in [19], hardened cement paste was observed even in the pores of LWA that was initially pre-saturated. Nevertheless, in the latest case, the effect of tightening the lightweight aggregate external shell was less pronounced and it was of secondary importance due to the much weaker interfacial transition zone.

The idea of the impregnation of lightweight aggregates [22,24] or recycled concrete aggregates (RCA) [21,22,23,26] with cement paste seems to be less popular than pre-coating the aggregates with polymers [21,24,27,28,29,30,31,32]. There are probably some concerns regarding less effective impregnation with cement paste in concrete tightness enhancement, higher increase in density, or slightly longer procedure of preparation. Meanwhile, the impregnation with cement paste may lead to better compatibility with the cement matrix in composite, a stronger bond, lower susceptibility to segregation, and more strengthening of the aggregate being the weakest element of LWAC. Unfortunately, there is little comparative research on the effectiveness of various impregnants used for the pre-coating of lightweight aggregates. The examples of such valuable research are [24,25], focused on the comparison of effectiveness of impregnation of natural organic lightweight aggregate with different materials. It was proved that impregnation of LWA with cement paste may be more effective in the reduction of concrete water absorption in comparison to pre-coating the lightweight aggregate with sodium silicate solution, white latex solution, and wood oil. Only impregnation with a waterproof agent solution resulted in the lower water absorption of concrete. Concerning the compressive strength and modulus of elasticity enhancement, the results of LWA pre-coating with cement paste and with waterproof agent solution were comparable and better than those achieved for the other treatments. However, the durability of concrete with aggregate impregnated just with cement paste, exposed by magnesium sulfate solution, turned out to be superior to the composites prepared with LWAs treated with all other substances. In the case of application of cement paste for coating, its composition may consist of plain cement grout or a mixture of different cementitious materials or other minerals (i.e., cement, ground granulated blast furnace slag, ground colemanite, fly ash, silica fume) and admixtures (typically plasticizers or superplasticizers) [21,22,23,24,25,26].

The main goal of the research was to determine the impact of lightweight aggregate impregnation with cement paste on LWA properties. Additionally, the effect of aggregate tightening and strengthening due to impregnation with cement paste was also verified on concretes. In contrast to the tests discussed above, the aggregate pre-coating effect was analyzed more comprehensively, taking into consideration LWAs of different types and size. Moreover, impregnation was carried out with the use of cement pastes of various composition and rheological properties. Furthermore, different levels of initial LWA moisture content were considered in the research.

## 2. Materials and Methods

The research program was divided into two stages. The first stage was focused on tests of impregnated aggregate to establish the effect on LWA pre-coating on its properties. The second stage of the research was dedicated to the influence of LWA impregnation on concrete composite properties.

### 2.1. Pre-Coating of Lightweight Aggregates with Cement Paste and Their Tests

**Figure 1:**
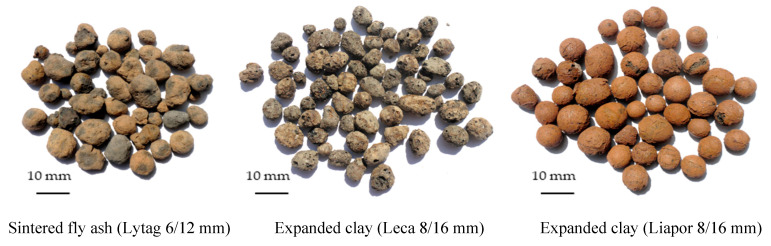
Types of lightweight aggregates used for the research.

To access the effect of LWAs tightening and strengthening due to their pre-coating with cement paste, three types of lightweight aggregates were selected: sintered fly ash Lytag, expanded clay Leca, and, additionally for comparison purposes, expanded clay Liapor (Figure 1). Two fractions of Lytag and Leca were used in the tests. In the case of reference Liapor, only the fraction 8/16 mm was considered. Basic properties of all five LWA fractions used for the research are presented in Table 1. Particle density and water absorption, bulk density, and crushing resistance were specified, respectively, according to the European Standards: EN 1097-3 [44], EN 1097-6 [45], and EN 13055-1 [46]. Porosity of aggregates was estimated on the basis of particle density and specific density determined by pycnometer method. Besides the typical determination of water absorption after 24 h, for each aggregate, the development of water absorption in time after 2 min until its stabilization was assessed. The aggregates selected for the research differed considerably in porosity and pore structure. Lytag was characterized by much lower porosity in comparison to both expanded clay aggregates. However, for this aggregate, the pore structure of the external shell and particle interior differed much less than in the case of the other aggregates. Therefore, it revealed more than twice higher water absorption than Liapor aggregate with a strongly sintered external shell. Meanwhile, Leca aggregate was characterized by the highest porosity and water absorption. On the one hand, the size of pores in the particle interior of Leca and Liapor was much bigger (up to 2 mm) in relation to Lytag (up to 0.2 mm). On the other hand, Leca showed a more porous external shell with visible surface pores in contrast to Liapor or even to Lytag.

Three cement pastes of different compositions were used for LWA impregnation (Table 2). They varied with water-cement ratio (w/c) and application of superplasticizer. All cement pastes were prepared with Portland cement CEM I 42.5 R and tap water. Additionally, for cement paste 2, a superplasticizer (Sika ViscoCrete 3) was dosed to achieve the consistency characteristic of the plain cement paste 1 with w/c = 0.55. It should be noted that in practice, cement paste 3, with relatively low w/c and not modified with superplasticizer, should not be used as a cement matrix for LWAC due to poor workability. In this research, this cement paste was applied only for comparison purposes to assess the influence of matrix rheology on the effect of absorption of water/cement paste by LWA. Therefore, it was used only for expanded clay aggregates. The chemical composition of the cement and the sintered fly ash is given in Table 3.

**Figure 2:**
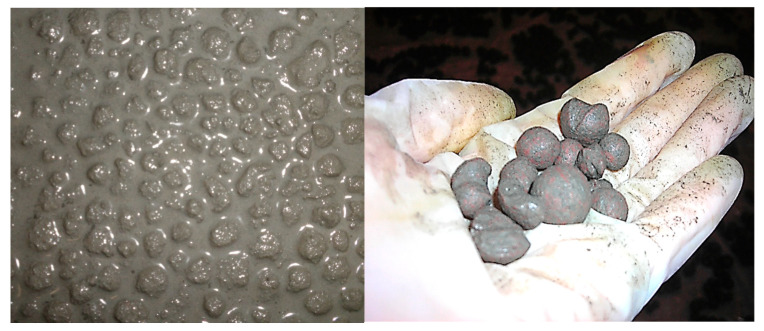
LWA immersed in cement paste (Lytag) and separated from the mixture (Liapor).

All lightweight aggregates were dried out before immersion in cement paste. Additionally, to check the influence of initial moisture content of LWA on the tightening and strengthening effects, Lytag aggregates were also used, pre-moistened to 17–18%. These levels of initial moisture content corresponded to LWA water absorption of fraction 4/8 mm and 6/12 mm, respectively, after 60 min. Th possibility of full saturation of the aggregate was excluded in the research program due to [8,9,16,17,18,19] reporting the significantly deteriorated performance of concrete with such pre-treated LWAs. Initially dried out or pre-moistened aggregates were added to the previously prepared cement paste and mixed in a concrete mixer for 5 min. In total, 17 different mixtures were prepared. Then, after 30 min, the aggregates were separated from the cement pastes with sieves with mesh 4 mm. Additionally, the excess of cement paste was removed from the LWA surface with a cloth. The time of this procedure was limited to 30 s in order to not absorb the water accommodated in the aggregate particles necessary for further cement paste hydration. The examples of LWA immersed in cement paste and LWA separated from the mixture are presented in Figure 2. After separation, the aggregates were stored on a sheet of foil to dry out for 24 h. Next, they were packed into bags. After 28 days, the aggregates were subjected to the following testes: water absorption, particle density, and crushing resistance according to EN 1097-3 [44], EN 1097-6 [45], and EN 13055-1 [46], respectively. Descriptions and parameters of all aggregates separated from cement pastes are presented in Table 4.

### 2.2. Preparation of Concretes with Impregnated LWAs and Their Tests

In order to verify the influence of LWA pre-coating on properties of lightweight composites, 8 different concretes were prepared. All concrete mixtures were made with Portland cement CEM I 42.5 R, the same applied for aggregate impregnation. As fine aggregate, natural sand was used, while as coarse aggregate, sintered fly ash (Lytag) and expanded clay (Leca) pre-coated with cement paste 1 (see Table 2) in dry state were selected. Additionally, for reference concretes as coarse aggregate plain, non-impregnated LWAs were used. All concretes were characterized by w/c = 0.55 and the same volume composition. Volume share of lightweight aggregate and cement matrix (mortar) in composites were 440 and 560 dm^3^/m^3^, respectively. Therefore, all concretes differed only by LWA mass content. Lightweight aggregates were dried out before application to concrete. To maintain suitable mixture workability and the same volume proportions, it was necessary to initially moistened non-impregnated LWAs used for references concretes. The target consistency for all fresh concretes was assumed as F4, tested and classified according to [47,48], respectively. The compositions of prepared concretes are presented in Table 5.

For each concrete, series nine cube specimens with sides of 100 mm were cast. After 24 h, all specimens were demolded and then stored in a climatic chamber (RH = 100%, T = 20 °C) for 27 days, according to EN 12390-2 [49]. Concrete specimens of each concrete series were subject to density and compressive strength tests, according to EN 12390-7 [50] and EN 12390-3 [51], respectively. The water absorption test, due to lack of suitable European Standard, was carried out according to Polish Standard PN-88/B-06250 [52]. Since European Standard procedures are well known, they are not described here. The procedure of the water absorption test was as follows: concrete specimens were saturated in water until their weights stabilized and their mass was determined. Then, the specimens were dried out at a temperature of 105 °C, and their oven-dried mass was specified. Water absorption was calculated as the water content in a saturated specimen related to the oven-dried specimen’s weight, expressed as a percentage.

## 3. Results

### 3.1. Results of Plain and Pre-Coated Aggregates Tests

#### 3.1.1. Assessment of Covering LWA with Cement Paste

**Figure 3:**
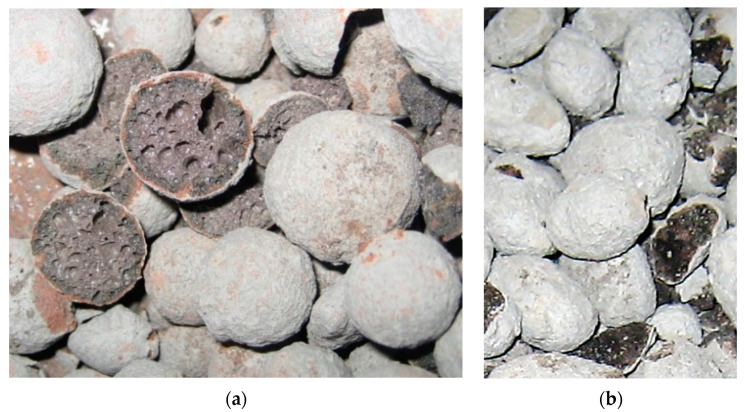
Expanded clay aggregates after crushing tests: (**a**) Liapor 8/16 mm, (**b**) Leca 8/16 mm.

**Figure 4:**
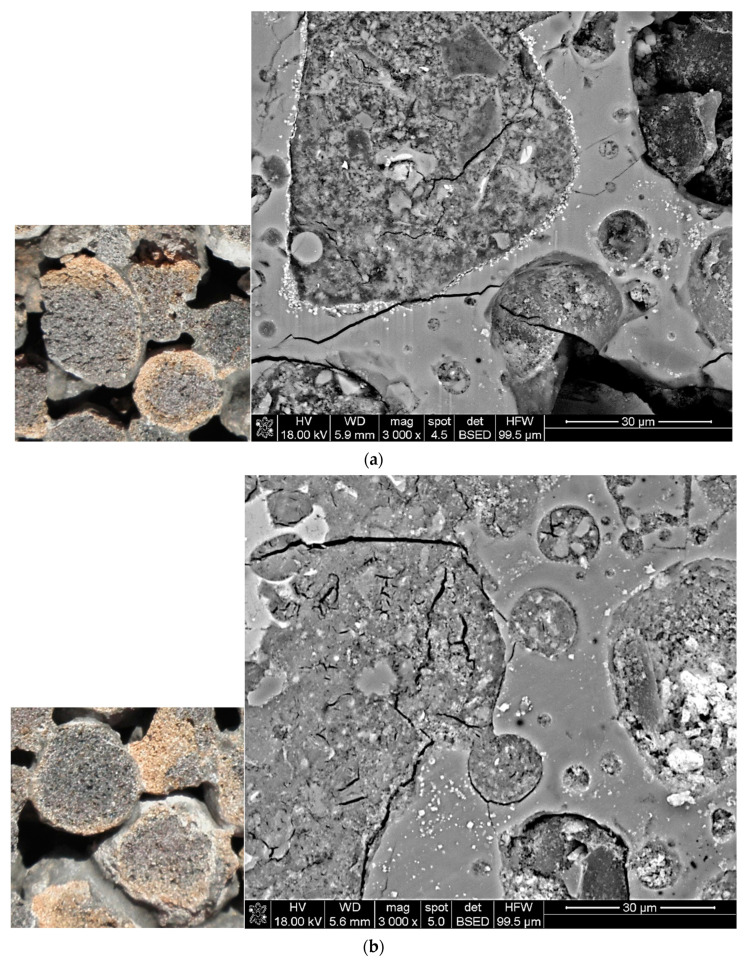
Cross-sections of coated particles of Lytag 6/12 mm and their shell pores filled with cement paste: (**a**) LWA initially moistened, (**b**) LWA initially dried out.

Thickness of cement paste covering LWA particles was assessed after crashing the samples of aggregates. For cement pastes 1 and 2, it ranged from 0.1 mm up to 0.3 mm. Greater cover thickness, even up to 1 mm, was observed for less liquid cement paste 3. In the case of all three LWA types, it seems that the cement paste coating only adhered to the LWA surface (Figure 3 and Figure 4). However, for Lytag and especially for Leca aggregates, characterized by a more porous external shell, the cement paste filled the surface pores forming a strong mechanical interlocking between the cover and the aggregate. This phenomenon was limited for Liapor aggregate due to the highly sintered external shell. The auxiliary analysis of aggregate particles under scanning electron microscope (SEM) in fact showed that the penetration of cement paste into LWA particles occurred in the case of all aggregates but on the microscale. Cement paste was able to reach micropores over 500 μm deep. Nevertheless, such pores were rarely observed in Liapor. The above qualitative observations are consistent with those made in [41,42].

The SEM analysis of LWA particles also confirmed the reports presented in [19]. The penetration of cement paste into the aggregate pores was observed independently of LWA initial moisture content. Images of shell pores of sintered fly ash aggregate initially dried and moistened, and filled with cement paste are presented in Figure 4. Therefore, the mechanism of cement paste penetration probably consists of mixing water previously accommodated in the aggregate pores with the dissolved cement paste.

#### 3.1.2. Particle Density of Plain and Pre-Coated Aggregates

**Figure 5:**
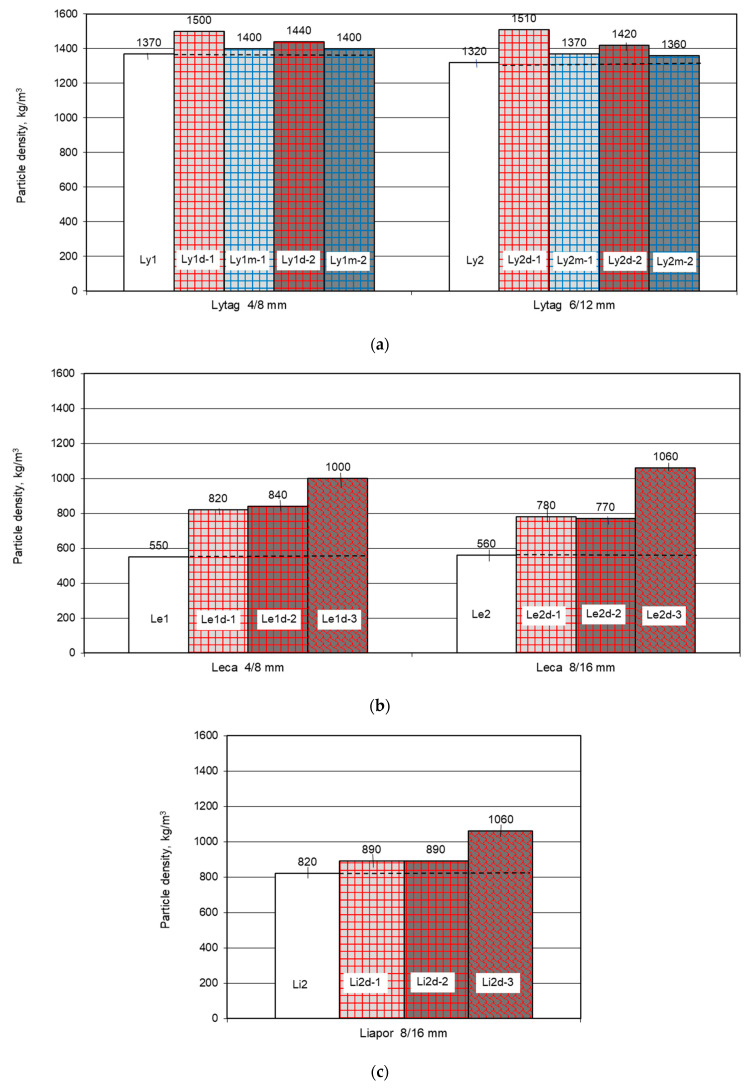
Mean oven dry particle density of lightweight aggregates, plain and impregnated with cement pastes: (**a**) Lytag, (**b**) Leca, (**c**) Liapor.

Particle density in oven dry condition for each plain LWA and LWA coated with cement paste was determined in three samples. The mean results of particle density for all 22 lightweight aggregates (plain as reference and impregnated with cement paste) are presented in Figure 5. For plain aggregates, the particle density ranged from 550 kg/m^3^ (for Leca aggregate) to 1370 kg/m^3^ (for Lytag aggregate). The density of LWAs immersed previously in cement paste was visibly higher and varied from 770 kg/m^3^ for Leca aggregate to 1510 kg/m^3^ for Lytag aggregate.

Individual results of particle density differed from the average value by no more than 20 kg/m^3^. Therefore, the coefficient of variation, defined as the ratio of a standard deviation and an average value for the particle density of the tested aggregates, was very low and ranged from 0 to 0.02.

#### 3.1.3. Crashing Strength for Plain and Pre-Coated Aggregates

**Figure 6:**
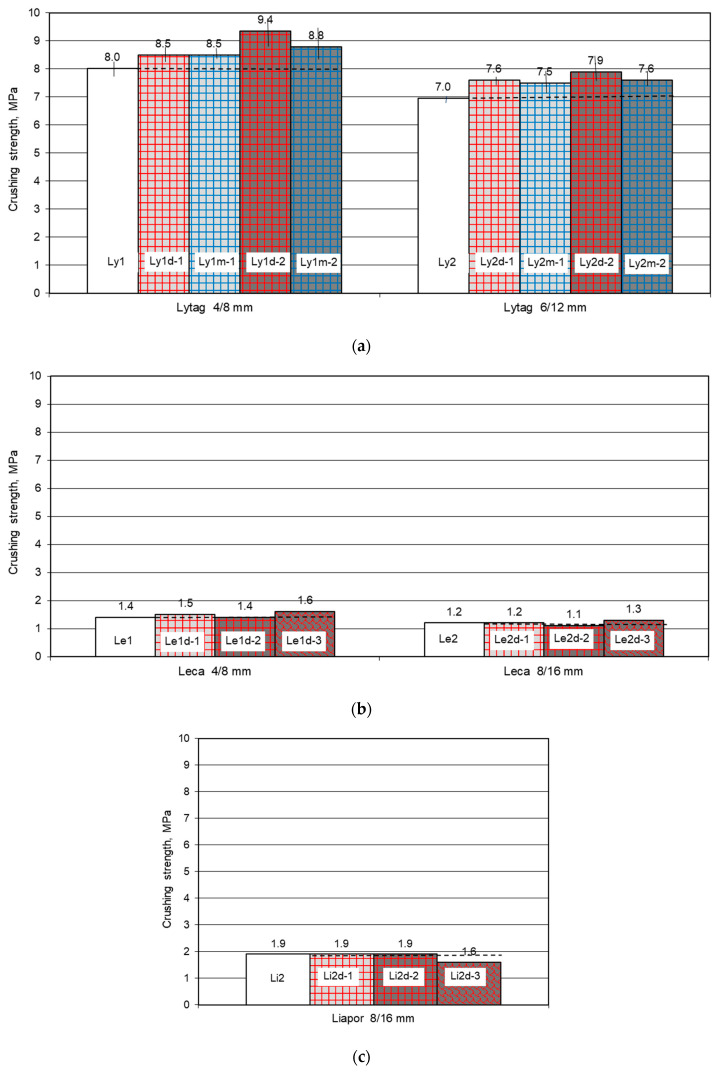
Mean crushing strength of lightweight aggregates, plain and impregnated with cement pastes: (**a**) Lytag, (**b**) Leca, (**c**) Liapor.

Crashing strength in oven dry condition for each plain LWA and LWA coated with cement paste was determined in three samples. The mean results of crushing strength for all 22 lightweight aggregates (plain as reference and impregnated with cement paste) are presented in Figure 6. For plain aggregates, the strength ranged from 1.2 MPa (for Leca aggregate) to 8.0 MPa for (Lytag aggregate). Crushing strength of LWAs immersed previously in cement paste was visibly higher only for Lytag aggregate and reached up to 9.4 MPa. For Leca aggregate, the strength increment due to impregnation in cement paste was inconsiderable, while in the case of Liapor no change in strength was observed at all.

Due to the specifics of the crushing test, the individual results differed from the average value, usually by 0 or 0.1 MPa. Only in 3 cases of 22 aggregates this difference was 0.2 MPa. For Lytag, much stronger than the other tested aggregates, such a difference corresponded to a very low coefficient of variation of crushing strength and equaled to 0–0.02. Meanwhile, for several times weaker expanded clay aggregates, this small result spread means a higher coefficient of variation: 0–0.09.

#### 3.1.4. Water Absorption and Its Development in Time for Plain and Pre-Coated Aggregates

**Figure 7:**
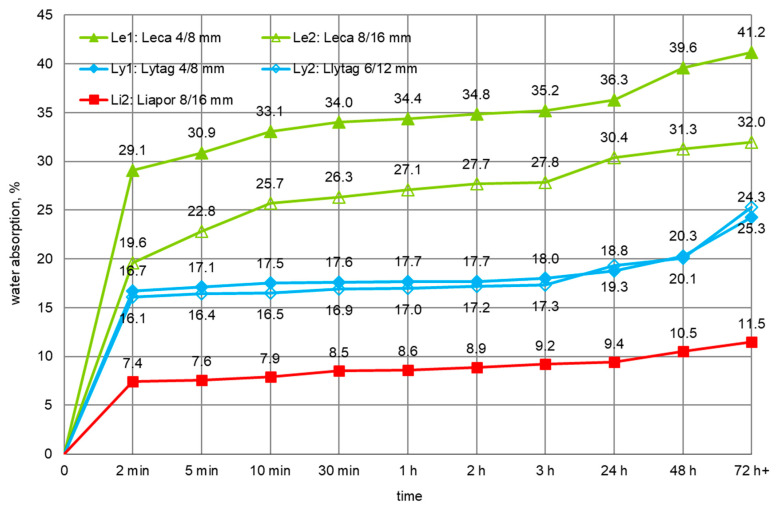
Development of water absorption of plain lightweight aggregates in time.

Water absorption for each plain LWA and LWA impregnated with cement paste was determined in three samples. The development of water absorption in time for plain aggregates was specified after immersion of LWA in water for 2, 5, 10, and 30 min, and 1, 2, 3, 24, 48, and 72 h. The mean values for the five plain aggregates are presented in Figure 7. The curves of water absorption development in time considerably differed for all LWA types and reflect their various porosity and pore structure. Leca 4/8 mm was characterized by the highest water absorption, 41.2%, while the bigger size Leca 8/16 mm, due to the more sintered shell, revealed a much lower value, 32.0%. Lytag aggregate showed moderate water absorption comparable for both fractions 4/8 and 6/12 mm, 24.3 and 25.3%, respectively. However, the water absorption of Liapor (11.5%) was more than twice smaller in comparison to Lytag and almost four times lower in relation to Leca.

**Figure 8:**
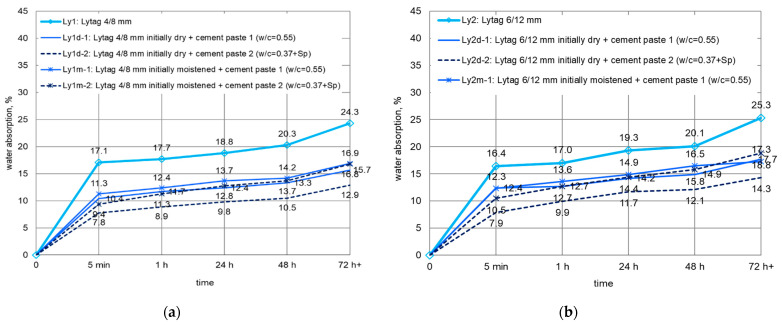
Development of water absorption in time for Lytag aggregate impregnated with cement pastes: (**a**) Lytag 4/8 mm, (**b**) Lytag 6/12 mm.

**Figure 9:**
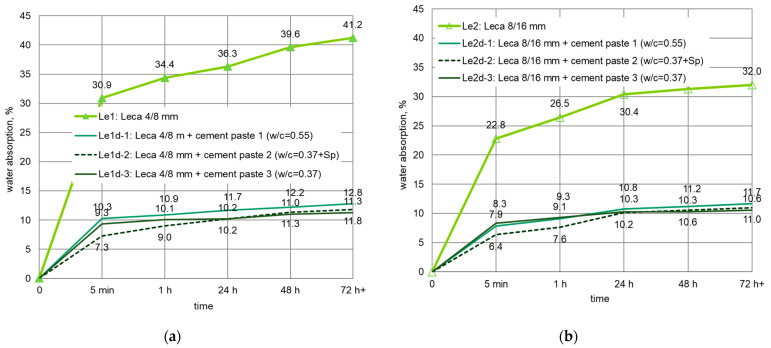
Development of water absorption in time for Leca aggregates impregnated with cement pastes: (**a**) Leca 4/8 mm, (**b**) Leca 8/16 mm.

**Figure 10:**
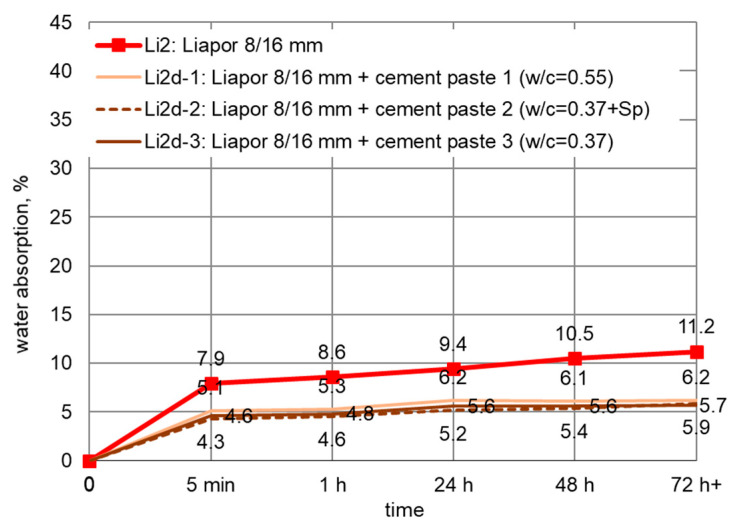
Development of water absorption in time for Liapor 8/12 mm impregnated with cement pastes.

For LWAs pre-coated with cement paste, the water absorption was determined after immersion in water for 5 min, and 1, 24, 48, and 72 h. The mean values of water absorption for different LWAs impregnated with cement pastes are presented separately for each aggregate fraction in Figure 8, Figure 9 and Figure 10. Penetration of cement paste into lightweight aggregates external shell significantly limited their water absorption. The biggest, with almost four times reduction in water absorption to 11.3%, was observed in the case of Leca 4/8 mm. However, the smallest water absorption (5.7%) was revealed by coated Liapor aggregate.

Individual results of water absorption differed from the average value by 0 to 1.1 percentage points. The coefficient of variation for water absorption of the tested aggregates was very low and ranged from 0 to 0.03.

### 3.2. Results of Hardened Concrete Tests

#### 3.2.1. Density of Concretes with Plain and Pre-Coated Aggregates

**Figure 11:**
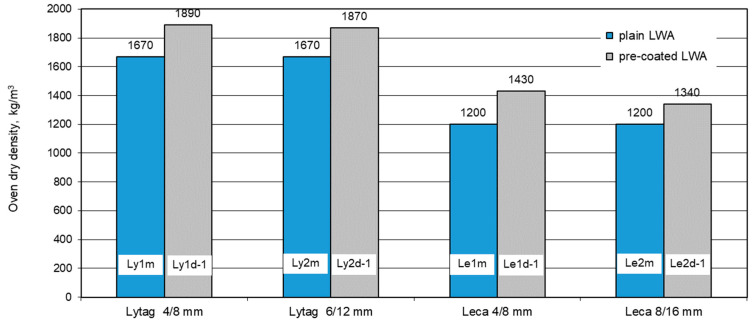
Mean oven dry density of concretes with plain and pre-coated lightweight aggregates.

Oven dry density for each LWAC was determined in three specimens used previously for water absorption tests. The mean results of the density for all eight composites (with plain aggregates as reference and with LWAs impregnated with cement paste) are presented in Figure 11. For reference concretes, the density ranged from 1200 kg/m^3^ (for Leca concrete) to 1670 kg/m^3^ (for Lytag concrete). The density of LWAC containing pre-coated aggregates was visibly higher and varied from 1340 kg/m^3^ for Leca concrete to 1890 kg/m^3^ for Lytag concrete.

Individual results of density differed from the average value by no more than 30 kg/m^3^. Therefore, the coefficient of variation of concrete density was very low and ranged from 0 to 0.02.

#### 3.2.2. Compressive Strength of Concretes with Plain and Pre-Coated Aggregates

**Figure 12:**
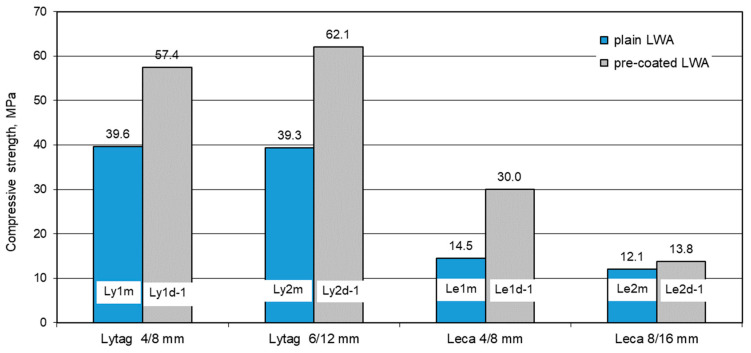
Mean compressive strength of concretes with plain and pre-coated lightweight aggregates.

Compressive strength of concretes for each LWAC was determined in six specimens. The mean results of the strength for all eight composites (with plain aggregates as reference and with LWAs impregnated with cement paste) are presented in Figure 12. For reference concrete, the strength ranged from 12.1 MPa (for Leca concrete) to 39.6 Mpa (for Lytag concrete). The compressive strength of LWAC containing pre-coated aggregates was significantly higher and varied from 13.8 Mpa for Leca concrete to 62.1 Mpa for Lytag concrete.

The coefficient of variation of concrete strength ranged from 0.01 to 0.06 and was slightly higher for concretes with Leca aggregate. However, all concretes made of impregnated aggregates revealed a smaller spread of results in comparison to results achieved in composites with plain aggregate.

#### 3.2.3. Water Absorption of Concretes with Plain and Pre-Coated Aggregates

**Figure 13:**
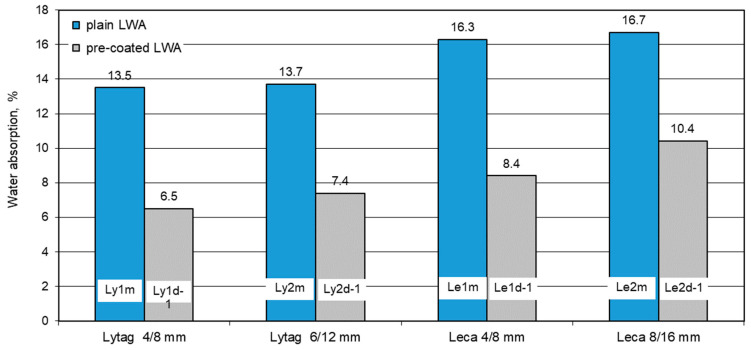
Mean water absorption of concretes with plain and pre-coated lightweight aggregates.

Water absorption of concretes for each LWAC was determined on three specimens. The mean results of the absorption for all 8 composites: with plain aggregates as reference and with LWAs impregnated with cement paste are presented in Figure 13. For reference concretes, the water absorption ranged from 13.5% (for Lytag concrete) to 16.7% (for Leca concrete). The water absorption of LWAC containing pre-coated aggregates was considerably lower and varied from 6.5% for Lytag concrete to 10.4% for Leca concrete.

Individual results of water absorption differed from the average value by no more than 0.1% point. Therefore, the coefficient of variation of the absorption was very low and ranged from 0 to 0.01.

## 4. Discussion

### 4.1. The Influence of Aggregates Pre-Coating on Their Properties

#### 4.1.1. Covering LWA with Cement Paste

Cement paste 3 turned out to be too viscous and formed a much thicker cover on LWA surface, in comparison to cement pastes 1 and 2. In the results, the properties of Leca and Liapor aggregates covered with cement paste 3 did not reflect the performance of the aggregates themselves in concrete, but performance of the aggregate together with the matrix cover. Therefore, it was not taken into consideration for Lytag aggregate tests at all. In the case of Leca and Liapor, tested earlier than Lytag, the results achieved for cement paste 3 may be treated only as informative. Since they are not representative and reliable for the aim of this research, they should be excluded from further quantitative analyses.

#### 4.1.2. Particle Density of Plain and Pre-Coated Aggregates

As it was expected, the immersion of lightweight aggregates in cement paste resulted in a particle density increase.

The analyses of initially dry lightweight aggregates showed that the biggest increase in particle density was observed in the case of Leca aggregate, since it was the lightest among the tested aggregates. Excluding the results for cement 3, the absolute and percentage increase in Leca density was as high as 210–290 kg/m^3^ and 38–53%, respectively. These density increments, although seeming to be big, are comparable as achieved for LWAs impregnated with polymers [27]. The lowest percentage increase in particle density (by 2–14%) was revealed by Lytag aggregate, as it was initially the densest LWA. Meanwhile, the lowest absolute increase in particle density (by 70 kg/m^3^) was specified for Liapor aggregate due to its tightest external shell.

Comparison of density results of Lytag immersed in cement pastes 1 and 2 of similar consistency but different water-cement ratio, indicated better penetration by cement paste 1 of a higher w/c. This is probably the reason of the visibly bigger density of Lytag impregnated with cement paste 1 than 2. However, this effect was not observed for expanded clay aggregates, in the case of Leca, due to bigger pores in the external shell, being more available for both pastes, and in the case of Liapor, due to too tight shell, being less available for both cement pastes.

Initial moistening of LWA limited its absorption of water and cement paste. In effect, for initially moistened Lytag aggregates (Ly1m-1, Ly1m-2, Ly2m-1, Ly2m-2t), the particle density increase was as low as 2–4% (by 30-50 kg/m^3^). The value of this increase corresponds to that revealed in [43] for very tight lightweight aggregates.

The influence of LWA size on particle density increase was of secondary importance to the initial density of the plain aggregate. That is why after immersion in cement paste, Lytag 6/12 mm showed a bigger density increase in comparison to the smaller size and more dense Lytag 4/8 mm. Meanwhile, Leca 8/16 mm revealed a lower increment in particle density when compared with the smaller size but less dense Leca 4/8 mm.

#### 4.1.3. Crashing Strength for Plain and Pre-Coated Aggregates

Due to the extremely porous structure of the tested expanded clay aggregates (porosity ranged 71–80%), the effect of tightening their external shell was not able to visibly increase the strength of the whole LWA particles. Furthermore, in the case of Liapor aggregate, characterized by the strongly sintered external shell, filling a relatively small number of pores with cement paste was irrelevant due to the high initial stiffness of the shell. Meanwhile, for Lytag aggregate, the effect of the external shell tightening was sufficient to make it stiffer and pronouncedly affect the strength of the whole aggregate particles. As a result of impregnation of Lytag aggregate with cement paste, its crushing strength increased by 6 up to 17% in comparison to plain particles. The increase was dependent on all three parameters taken into consideration: aggregate size, aggregate initial moisture content, and cement paste strength.

Application of stronger cement paste for immersion of Lytag resulted in its stronger stiffening effect. Initially dry aggregate coated with cement paste 2 revealed a crushing strength that was higher by 17 and 14%, respectively, for fractions 4/8 mm and 6/12 mm, while impregnation with weaker cement paste 1 gave a result higher by 6 and 9%. For aggregates initially moistened, the effect was less pronounced. On the one hand, the weakened absorption mechanism was not able to provide such a good tightening of external shale as initially dry aggregates. On the other hand, due to the large amount of water accommodated in the initially moistened LWA particles, the real water-cement ratio of cement paste filling the external pores of the aggregate was probably higher than the ratio of cement paste present in the pores of initially dried aggregate.

#### 4.1.4. Water Absorption and Its Development in Time for Plain and Pre-Coated Aggregates

When assessing the development of water absorption of plain lightweight aggregates (Figure 7), it should be stated that during the first minutes all tested aggregates were able to absorb most of the water. For such aggregates as Leca and Liapor, characterized by the bigger difference in porosity structure between the external shell and particle interior, the ratio of water absorption after 2 min and 1 h was 72–86%. This range corresponds to the values determined in [3,12,14]. However, in the case of tested Lytag of a less diversified structure, the ratio was as high as 94–95%.

The most pronounced effect of tightening the external shell with cement paste was observed in the case of water absorption. Penetration of cement paste into the surface pores of initially dry LWAs resulted in a considerable reduction in their water absorption. In the case of Leca 4/8 mm, characterized by the most open structure, the decrease in water absorption was on average 71%. Leca 8/16 mm with initially lower water absorption showed a mean reduction of 66%. Corresponding decreases in water absorption for Liapor 8/12 mm, Lytag 4/8 mm, Lytag 6/12 mm were on average 43, 42, and 35%, respectively. It should be noted that the reduction in water absorption for all tested aggregates seemed to be slightly higher at the earlier time of test. Moreover, the penetration of cement paste into LWA external shell limited the development of water absorption in time. Percentage increments of water absorption in time intervals were lower for lightweight aggregates coated with cement paste in comparison to plain LWAs.

Generally, the better sealing effect was observed when lightweight aggregates were impregnated in stronger cement paste. The most advantageous reduction was achieved for aggregates immersed in cement paste 2. Cement paste 3 of the same w/c but without superplasticizer, as well as cement paste 1 of higher w/c were not able to seal LWA structures so effectively. Nevertheless, due to the different pore structures of expanded clay and sintered fly ash aggregates, the quantitative influence of the cement paste type was different for various LWA types. For Lytag aggregate coated with various cement pastes, the reduction in water absorption clearly differed (Figure 8). For example, the decrease for Ly1d-1 and Ly2d-1 was, on average, 35 and 26%, respectively, while for Ly1d-2 and Ly2d-2, it equaled, on average, to 49 and 43%, respectively. Meanwhile, in the case of Leca and Liapor aggregate (Figure 9 and Figure 10), the influence of applied cement paste type was definitely less pronounced.

Initial moistening of Lytag limited the effect of aggregate sealing with cement paste in comparison to this observed for initially dry LWA. Pre-moistened Lytag coated with cement paste showed a visibly lower reduction in water absorption. It ranged from 23–35%, and, the same as for dry aggregates, it was higher for stronger cement paste and for smaller size Lytag.

The sealing effect of lightweight aggregates with cement paste turned out to be stronger for smaller size aggregates. Although the cement paste penetration depth was comparable for both fractions of Lytag and Leca aggregate, the share of sealed shell in LWA particle volume was higher for fraction 4/8 mm than for the bigger size.

The comparison of the achieved results for coated Lytag aggregate with the results reported in [9], related to water absorption of concretes made of the same aggregate and cement matrices of the same water-cement ratio, indicated that the effect of lightweight aggregate sealing may play a dominant role in the reduction of concrete water absorption. The decrease in cement matrix w/c, caused by the absorption of mixing water by the aggregate in concrete, is of less importance.

It should be noted that the effectiveness of the tested LWAs tightening with cement paste turned out to be comparable to this achieved by impregnating LWAs with polymers. The research dedicated to pumice aggregate pre-coated with different polymers [27,32], characterized by water absorption similar to the tested Leca or Lytag aggregates, showed a comparable reduction in water absorption.

### 4.2. The Influence of Aggregates Pre-Coating on Concrete Properties

#### 4.2.1. Density of Concretes with Plain and Pre-Coated Aggregates

All concretes made with pre-coated aggregates revealed higher density than corresponding reference concretes. The density increase for concretes with both Lytag aggregates, 4/8 and 6/12 mm, as well as with Leca 8/16 mm was 12%, while in the case of concrete with Leca 4/8 mm it was as high as 19%. This greater increase in density corresponds to the greatest increase in particle density for Leca 4/8 mm after impregnation with cement paste.

**Figure 14:**
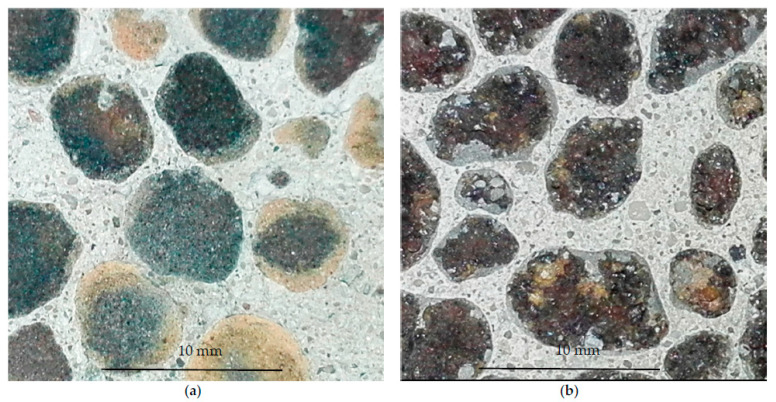
Appearance of a fracture in lightweight concretes with impregnated aggregates (**a**) Lytag 4/8 mm (Ly1d-1) and (**b**) Leca 4/8 mm (Le1d-1), subject to compression.

The difference in appearance of a fracture of lightweight concretes with impregnated aggregates Lytag and Leca of the same fraction is presented in Figure 14. In the case of Lytag concrete, LWA sintered shell prevented the visible access of the cement paste into its structure, while for Leca concrete, bigger pores filled with cement paste are clearly visible.

The observed density increment of concretes prepared with aggregates pre-coated with cement paste was greater than indicted in [24] for concretes with impregnated LWAs, or in [26,33] for concretes made of impregnated RCAs due to significantly lower initial water absorption of those aggregates. However, the achieved increase in density was similar or lower when compared to concretes with aggregates of higher initial water absorption, even when they were pre-coated with polymers [29].

#### 4.2.2. Compressive Strength of Concretes with Plain and Pre-Coated Aggregates

Compared to reference concretes, the application of pre-coated both fractions of Lytag aggregate, 4/8 mmm and 6/12 mm, as well as Leca 4/8 mm resulted in a considerable increase in compressive strength: 45, 58, and 107%, respectively. Meanwhile, impregnation of Leca 8/16 mm with cement paste resulted in only a 14% increase in strength. In this case, tightening external layers of the aggregate could not lead to pronounced strength enhancement when the remainting parts of aggregate particles were still characterized by a very large number of pores. For smaller fractions of Leca aggregate, the proportion between a thickness of tightened external cover and the diameter of non-impregnated particle parts was much greater. As a result, the effectiveness of LWA pre-coating with cement paste turned out to be the most visible for the concrete, showing the biggest increase in density.

Obtained compressive strength increase results for concretes prepared with LWAs pre-coated with cement pastes are qualitatively consistent with the increments of crashing strength observed for the impregnated aggregates, that were discussed in Section 4.1.3. Nevertheless, quantitative assessment showed greater effectiveness of LWAs impregnation on concrete properties than on aggregate properties themselves.

Excluding Leca 8/16 mm, the strengthening effect of impregnation of the other applied LWAs with cement paste on concrete should be assessed as much more pronounced than the effect mentioned in the research described in [24,26,29,32,33,37,40], regardless of the types of aggregate and impregnating substance.

It is worth emphasizing that due to suitable material compatibility of LWAs, cement paste cover, and cement matrix in concrete, the applied impregnation of lightweight aggregates did not deteriorate the adhesion of the matrix to the aggregates. As a result, all concrete specimens under load revealed the fracture path through the aggregate particles instead of the bond (Figure 14).

#### 4.2.3. Water Absorption of Concretes with Plain and Pre-Coated Aggregates

All tested concretes made of pre-coated aggregates showed a remarkable decrease in water absorption by up to 52% in relation to the reference composites. The most effective limitation of water absorption was observed for concretes with fractions 4/8 mm. The lowest reduction in water absorption (by 38%) was revealed by concrete with pre-coated Leca 8/16 mm. Although the impregnation of this LWA did not lead to a significant increase in concrete strength, the treatment was sufficient to visibly limit the water absorption of the composite.

The decrease in water absorption revealed that concretes prepared with LWAs pre-coated with cement pastes differed qualitatively and quantitatively from the reduction in water absorption observed for the impregnated aggregates, discussed in Section 4.1.4. Impregnation of Leca fractions led to the much higher reduction in water absorption of the aggregates in comparison to Lytag fractions, while pre-coating of Lytag LWAs was much more effective for the decrease in water absorption of concretes compared to Leca aggregates.

The research [35] showed that the application of polymers for LWA impregnation may be even more effective for the decrease in water absorption of concrete than it was proved for the tested concretes made of aggregates pre-coated with cement paste. Nevertheless, the testing procedure for that research was different and the water absorption was observed only for 24 h. In other tests [24,26], the tightening effect of aggregate impregnation, even when polymers were used, seemed to be less pronounced than the effect revealed in this research.

## 5. Conclusions

The analysis of the archived test results showed that pre-coating of lightweight aggregates with cement paste may enhance both the properties of LWAs themselves, and properties of concrete made of these aggregates. In particular, the research results provide the basis for the following main conclusions:

- The effectiveness of strengthening and tightening of lightweight aggregate by its impregnation with cement paste is mainly dependent on the aggregate type, especially on its porosity and pore structure. The other parameters analyzed in this research, i.e., aggregate size, strength and rheology of cement paste, initial LWA moisture content, were of less importance.
- The most pronounced influence of LWA impregnation with cement paste was observed for water absorption. The surface tightening effect resulted in a significant reduction in water absorption of all five tested aggregates. The aggregates coated with cement paste revealed water absorption 1.8–3.7 times lower than for plain LWAs.
- Verification of the influence of pre-coating of selected LWAs with cement pastes on the concrete properties proved that the aggregate impregnation is a very effective method to enhance concrete properties. Although the application of pre-coated aggregates resulted in a higher concrete density (by up to 19%), the strength increase (by up to 107%) and the water absorption reduction (by up to 52%) were much more pronounced.

To sum up, assessment of the properties of LWAs pre-coated with cement paste and their suitability for concrete showed the purposefulness of the application of such a treatment, in order to reduce water content in fresh concrete necessary to maintain target workability, as well as to tighten and strengthen hardened concrete. Pre-coating of lightweight aggregates with cement paste turned out to be an advantageous alternative to impregnation of aggregates with polymers. This research indicated that the application of LWA impregnation may be advisable for aggregates with a structure similar to the tested Lytag or Leca, provided that its size is limited. However, such an initial LWA preparing seems to be pointless for aggregates with tight structure, as with the tested Liapor.

Due to the fact that the verification of the impact of lightweight aggregates impregnation on concrete properties was limited only to composites made of aggregates pre-coated with one cement paste type, further research should be carried out taking into account the LWAs impregnation with cement pastes of different rheological properties and strengths. Additionally, the possibility of using mineral additives to cement slurries applied for pre-coating should be considered, which would enable the reduction in both the cost and the carbon footprint of concrete production.

## Figures and Tables

**Table 1:** Properties of lightweight aggregates used in the research.

LWA Type	Fraction	LWA Designation	Particle Density, kg/m^3^	Bulk Density, kg/m^3^	Porosity, %	Water Absorption after 24 h, %	Max. Water Absorption, %	Crushing Resistance, MPa
Lytag	4/8 mm	Ly1	1320	730	47	18.8	24.3	8.0
Lytag	6/12 mm	Ly2	1340	720	46	19.3	25.3	7.2
Leca	4/8 mm	Le1	550	330	80	36.4	41.2	1.4
Leca	8/16 mm	Le2	560	310	80	30.7	32.0	1.2
Liapor	8/16 mm	Li2	780	460	71	9.4	11.5	1.9

**Table 2:** Parameters of cement pastes used for LWA immersion.

Cement Paste Designation	Water-Cement Ratio (w/c)	Superplasticizer, in% of Cement Mass
1	0.55	0
2	0.37	1.0
3	0.37	0

**Table 3:** Chemical composition of the cement and the sintered fly ash.

Component	CaO, %	SiO_2_, %	Al_2_O_3_, %	Fe_2_O_3_, %	SO_3_, %	MgO, %	Na_2_O_eqv_., %	Loss of Ignition, %
CEM I 42,5R	63.6	22.1	5.6	3.1	2.6	1.2	0.8	0.9
Lytag	2.2	58.0	22.0	3.1	0.3	1.4	0.9	<4

**Table 4:** Description of impregnated and non-impregnated LWAs used in the research.

N^o^	LWA Designation	LWA Type	LWA Fraction	LWA Moisture Content, %	Cement Paste Type
1	Ly1d	Lytag	4/8 mm	0	-
2	Ly1d-1	Lytag	4/8 mm	0	1
3	Ly1d-2	Lytag	4/8 mm	0	2
4	Ly1m-1	Lytag	4/8 mm	17	1
5	Ly1m-2	Lytag	4/8 mm	17	2
6	Ly2d	Lytag	6/12 mm	0	-
7	Ly2d-1	Lytag	6/12 mm	0	1
8	Ly2d-2	Lytag	6/12 mm	0	2
9	Ly2m-1	Lytag	6/12 mm	18	1
10	Ly2m-2	Lytag	6/12 mm	18	2
11	Le1d	Leca	4/8 mm	0	-
12	Le1d-1	Leca	4/8 mm	0	1
13	Le1d-2	Leca	4/8 mm	0	2
14	Le1d-3	Leca	4/8 mm	0	3
15	Le2d	Leca	8/16 mm	0	-
16	Le2d-1	Leca	8/16 mm	0	1
17	Le2d-2	Leca	8/16 mm	0	2
18	Le2d-3	Leca	8/16 mm	0	3
19	Li2d	Liapor	8/16 mm	0	-
20	Li2d-1	Liapor	8/16 mm	0	1
21	Li2d-2	Liapor	8/16 mm	0	2
22	Li2d-3	Liapor	8/16 mm	0	3

**Table 5:** Compositions of lightweight concretes prepared with impregnated and non-impregnated LWAs.

N^o^	Mix Designation	LWA Type	LWA Fraction	Cement Paste Used for LWA Pre-Coating	LWA, kg/m^3^	Water to LWA, kg/m^3^	Natural Sand, kg/m^3^	Cement, kg/m^3^	Water, kg/m^3^
1	Ly1m	Lytag	4/8 mm	-	603	109	512	420	231
2	Ly1d-1	Lytag	4/8 mm	1	660	-	512	420	231
3	Ly2m	Lytag	6/12 mm	-	581	99	512	420	231
4	Ly2d-1	Lytag	6/12 mm	1	660	-	512	420	231
5	Le1m	Leca	4/8 mm	-	243	80	512	420	231
6	Le1d-1	Leca	4/8 mm	1	275	-	512	420	231
7	Le2m	Leca	8/16 mm	-	246	62	512	420	231
8	Le2d-1	Leca	8/16 mm	1	343	-	512	420	231

## Data Availability

Not applicable.
